# THE IMPACT OF COVID-19: IDENTIFYING WELLNESS STRATEGIES FOR ACADEMIC GERONTOLOGY AND NURSING FACULTY AND STAFF

**DOI:** 10.1093/geroni/igad104.2607

**Published:** 2023-12-21

**Authors:** Katarina Friberg-Felsted, Cheryl Armstrong, Jennifer Macali, Sara Simonsen, Jennifer Clifton

**Affiliations:** University of Utah, Salt Lake City, Utah, United States; University of Utah, Salt Lake City, Utah, United States; University of Utah, Salt Lake City, Utah, United States; University of Utah, Salt Lake City, Utah, United States; University of Utah, Salt Lake City, Utah, United States

## Abstract

The continually evolving nature of the COVID-19 pandemic has presented challenges for individuals working in all sectors of the population, and academia is no exception. The nursing workforce is aging: the average age of a nurse is 52; 55% of the RN workforce is age 50 or older, and the average age of an employed nursing faculty is 46 years old. To date, little is known about the impact of the pandemic on individuals working in gerontology or nursing academia. The purpose of this study was to determine the impact of the COVID-19 pandemic on faculty and staff in a College of Nursing (CON) with a Gerontology program and identify practical strategies to promote health and wellness. A mixed-method survey was administered to faculty (n=140) and staff (n=139) in a College of Nursing in the Intermountain West. Quantitative and qualitative data were collected through a convenience sample. The researchers asked respondents to rate their experiences working from home, including the effects on work-related factors and social-lifestyle factors. Descriptive statistics were used to analyze the data. A total of 139 respondents completed the survey (faculty, n=86; staff, n=53). Respondents identified areas that were better and worse with regard to both work-related and social-lifestyle factors. Further, practical strategies to promote health and wellness were also identified. Finally, themes regarding support from leadership were recognized. This presentation will discuss these three results. These findings highlight the pandemic impact on aging nursing faculty and staff and introduce valuable strategies for promoting health and wellness.

